# Vegetative plaque on the left superior eyelid

**DOI:** 10.1002/ski2.277

**Published:** 2023-08-07

**Authors:** Colin G. Wikholm, Sach Thakker, Pegah R. Bakhshi, Adam J. Swigost, Alan Moshell, Michael A. Cardis

**Affiliations:** ^1^ Department of Dermatology MedStar Washington Hospital Center / Georgetown University Hospital Washington Columbia USA; ^2^ Georgetown University School of Medicine Washington Columbia USA

## Abstract

Herein we present case report of a 73‐year‐old female who developed a rapidly growing, ulcerated lesion on her left superior eyelid. Despite treatment for suspected infection, symptoms only marginally improved. Physical examination revealed a diffusely ulcerated multinodular tumour with overlying haemorrhagic and serosanguineous exudate. A shave biopsy led to a diagnosis of primary cutaneous anaplastic large cell lymphoma (pcALCL), a rare CD30+ lymphoproliferative disorder. The patient had no extracutaneous involvement on PET‐CT and her prognosis is good given the indolent nature of pcALCL. Differential diagnoses included merkel cell carcinoma, periocular sebaceous carcinoma, lymphomatoid papulosis, and extranodal natural killer/T cell lymphoma. Prognosis for pcALCL is generally good. Treatment recommendation for pcALCL is surgical excision with negative margins for localised disease, while intravenous brentuximab vedotin is suggested for widespread, relapsed, and refractory disease.

## Graphical abstract

We describe a 73‐year‐old female patient presenting with a vegetative plaque on her left super eyelid. She was initially misdiagnosed and erroneously treated with antibiotics prior to her referral to our dermatology clinic.

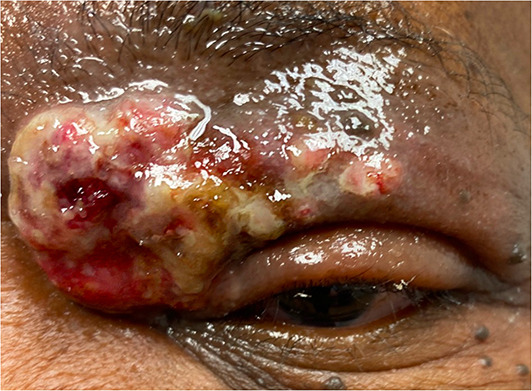

## 1 CASE REPORT

A 73‐year‐old female with a past medical history of hypertension, hyperlipidaemia, diabetes, chronic kidney disease, hypothyroidism, and cataracts presented with a vegetative lesion on her left superior eyelid. The lesion appeared 2 weeks prior as an asymptomatic papule which grew quickly and proceeded to ulcerate and bleed. Ophthalmology consultation yeilded a preliminary diagnosis of infection and the patient was prescribed gentamicin, mupirocin, and clindamycin with only minor improvement of symptoms.

**FIGURE 1:**
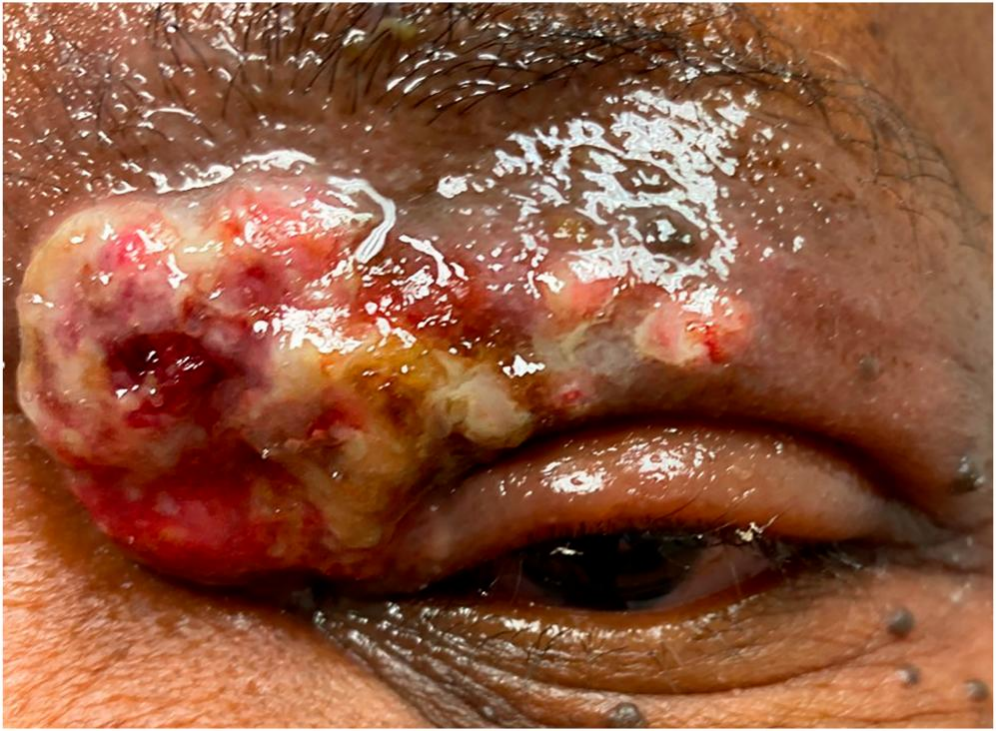
Vegetative lesion on the left superior eyelid. The epidermal surface is tan‐white, elevated, granular, and crusted.

Physical examination revealed an exophytic and poorly demarcated diffusely ulcerated multinodular tumour with overlying haemorrhagic and serosanguineous exudate that extended from the left medial canthus to the mid‐pupillary line (Figure 1). Workup for extracutaneous involvement was negative on positron emission tomography–computed tomography (PET–CT).

**FIGURE 2:**
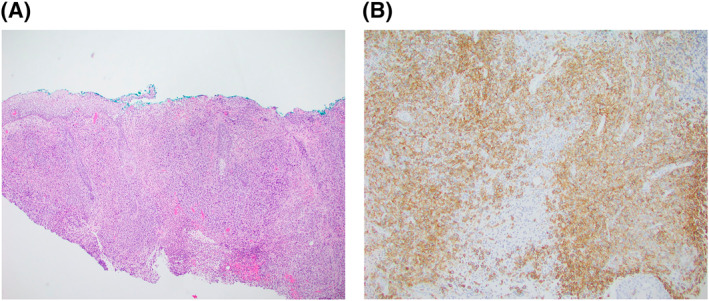
Histology of the specimen on a haematoxylin‐​eosin stain (a) and with CD30 immunohistochemistry (b).

**FIGURE 3:**
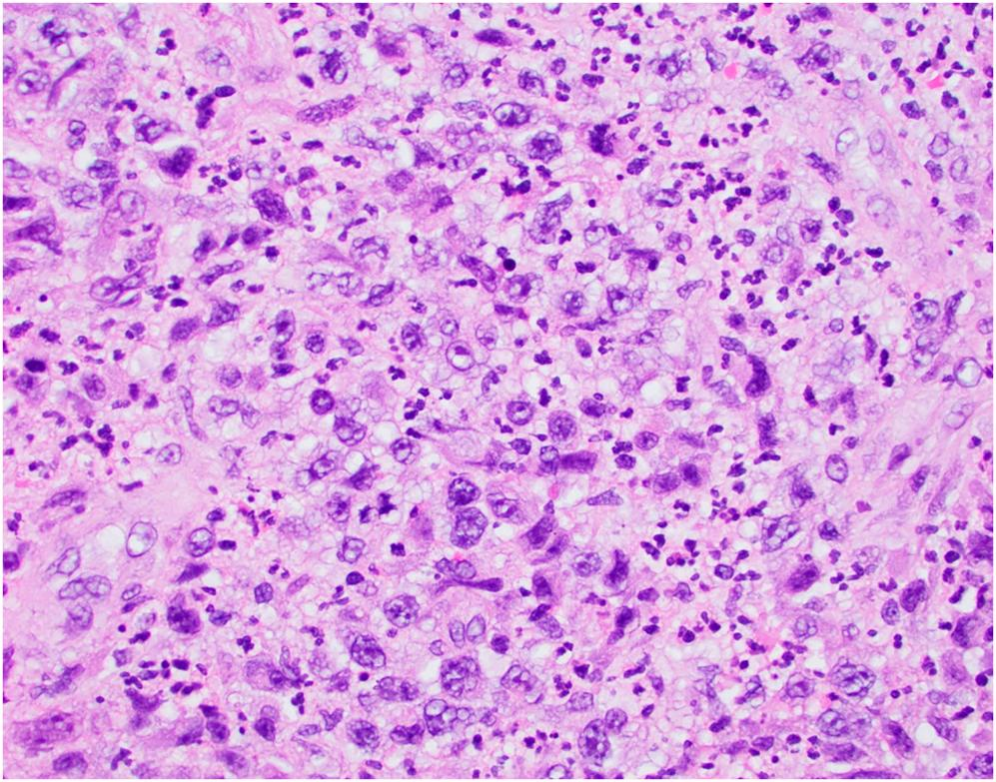
High power view of the proliferation featuring large, atypial and pleomophic lymphid cells with background inflammatory cells. Occasional mitotic figures are noted.

Shave biopsy revealed a dense dermal infiltrate of large, anaplastic CD30+ lymphocytes arranged in cohesive sheets (Figure 2a/2B) with scattered mitotic figures and mixed inflammatory cells including eosinophils (Figure 3). Numerous histiocytes, neutrophils, plasma cells, and lymphocytes were seen throughout the specimen along with suppurative and granulomatous inflammation. Microbial stains were negative. CD30 and CD45 stained almost 100% of the atypical lymphoid cells within the dermis. CD3 stained about 90% of the atypical lymphoid cells. ALK1, CK20, CD4, CD5, CD20, CD56, CD66, HSV‐1, HSV‐2, and VZV were negative. Pankeratin staining indicated a normal epidermis and hair follicle epithelium. SOX‐10 highlighted melanocytes in normal numbers within the epithelial portion of the specimen. Based on clinical and pathological correlation, a diagnosis of primary cutaneous anaplastic large cell lymphoma (pcALCL) was made.

## 2 DISCUSSION

Primary cutaneous anaplastic large cell lymphoma (pcALCL) is a chronic lymphoproliferative disorder that most commonly presents as a solitary or localised group of nodule(s) that may ulcerate.^1^ It represents up to 3% of cutaneous lymphomas, and may arise spontaneously or as a transformation of a preexisting lymphoma.^1^ There is typically a slight male predominance with pcALCL, and it is more commonly observed in individuals aged between 50 and 70. Additionally, pcALCL may be associated with immunosuppression (HIV/post organ transplantation).^2^ Overall extent of cutaneous lesions along with initial involvement of head/neck is associated with worse outcomes. Biopsy is often needed for diagnosis of pcALCL, which may otherwise be challenging or delayed due misdiagnosis of infectious, granulomatous, or autoimmune diseases.^1^, ^2^

Diagnosis requires CD30+ staining of atypical cells, although immunohistochemistry often shows a combination of T‐, B‐, mixed‐, or null‐phenotypes. Prognosis is good; the primary cutaneous form of the disease is relatively indolent, with around a 90% survival rate at 5 years^1^ The systemic form of ALCL is more aggressive and shows non‐localised disease on imaging. A mutation in the ALK gene is implicated in approximately 80% of cases of systemic anaplastic large cell lymphoma, and is less common in the localised form of the disease.^1^

Differentials for this diagnosis include merkel cell carcinoma, periocular sebaceous carcinoma, lymphomatoid papulosis, and extranodal natural killer/T cell lymphoma. Merkel cell carcinoma is a malignant, aggressive neuroendocrine tumour that most commonly presents as a rapidly enlarging lesion on sun exposed sites including the periorbital region.^3^ Histology reveals round blue cells that have large basophilic nuclei with a ground glass appearance, and a characteristic staining pattern defined by CK20 positivity in a perinuclear dot‐like pattern.^3^ Sebaceous carcinoma is a malignant, aggressive tumour that most commonly presents as a slowly enlarging lesion on the superior or inferior eyelid.^4^ Histology reveals a basaloid neoplasm with sebaceous differentiation, often with mitoses and comedonecrosis.^4^ Lymphomatoid papulosis, type D is a chronic lymphoproliferative disorder that most commonly presents as a waxing and waning papulonodular eruption.^1^ Histology reveals prominent epidermotropism with a CD8+ lymphocyte predominance.^1^ Lymphomatoid papulosis is clinically differentiated from primary cutaneous anaplastic large cell lymphoma by its waxing and waning papulonodular presentation. Extranodal natural killer/T cell lymphoma, nasal type is a malignant tumour that most commonly presents in the upper aerodigestive tract, but may rarely present with orbital involvement.^5^ Histology reveals a diffuse polymorphic infiltrate of atypical lymphoid cells and, in most instances, angiocentricity with numerous mitoses. The lesional cells are CD56+ and express cytotoxic markers. The clinical appearance is protean and the prognosis is poor.^5^

Surgical excision with negative margins is often the preferred treatment option for pcALCL presenting as solitary or localised disease, and is associated with complete remission rates of 100% and a recurrence rates of 40%.^6^ Intravenous brentuximab vedotin is the most appropriate treatment option for patients who present with widespread, relapsed, and/or refractory disease recalcitrant to methotrexate. In these patients, it is associated with an overall response rate of 75% and a complete remission rate of 31%.^6^

## AUTHOR CONTRIBUTIONS

**Colin G Wikholm**: Conceptualisation (lead); Investigation (lead); Methodology (lead); Project administration (lead); Writing – original draft (lead); Writing – review & editing (lead). **Sach Thakker**: Conceptualisation (equal); Investigation (equal); Methodology (equal); Project administration (equal); Writing – original draft (equal); Writing – review & editing (equal). **Pegah R. Bakhshi**: Investigation (equal); Methodology (equal); Writing – original draft (equal); Writing – review & editing (equal). **Adam J Swigost**: Conceptualisation (supporting); Writing – review & editing (supporting). **Alan N Moshell**: Conceptualisation (supporting); Writing – review & editing (supporting). **Michael A Cardis**: Conceptualisation (equal); Supervision (equal); Writing – review & editing (equal).

## CONFLICT OF INTEREST STATEMENT

None to declare.

## ETHICS STATEMENT

Not applicable.

## Data Availability

No data used in this study.
